# Coronavirus Disease Spread during Summer Vacation, Israel, 2020

**DOI:** 10.3201/eid2801.210177

**Published:** 2022-01

**Authors:** Ido Somekh, Eric A. F. Simões, Eli Somekh

**Affiliations:** Schneider Children’s Medical Center of Israel, Petah Tiqwa, Israel (I. Somekh);; Tel Aviv University, Tel Aviv, Israel (I. Somekh, E. Somekh);; University of Colorado School of Medicine, Aurora, Colorado, USA (E.A.F. Simões);; Mayanei Hayeshuah Medical Center, Bnei Brak, Israel (E. Somekh)

**Keywords:** COVID-19, SARS-CoV-2, severe acute respiratory syndrome coronavirus 2, viruses, respiratory infections, zoonoses, children, schools, summer vacation, Israel, *Suggested citation for this article*: Somekh I, Simões EAF, Somekh E. Coronavirus disease spread during summer vacation, Israel, 2020. Emerg Infect Dis. 2022 Jan [*date cited*]. https://doi.org/10.3201/eid2801.210177

## Abstract

The relative increase in coronavirus disease incidence during summer 2020 in Israel was most prominent in young children. This finding contrasts with the lower increase in incidence observed in children than in adults during the school attendance period. School closure without lockdown conditions might not be independently effective at reducing spread.

During July 1–August 31, 2020, the period of school vacation in Israel, coronavirus disease (COVID-19) cases surged and reached one of the highest rates in the world. We examined the nationwide involvement of children in this resurgence by comparing severe acute respiratory syndrome coronavirus 2 (SARS-CoV-2) infections by age dynamics during the period of school vacation with that during the school attendance period.

## The Study

We obtained data from public national data sources (*1*,*2*) (Appendix). To determine adjusted SARS-CoV-2 incidence rate ratios (IRRs), we adjusted age-specific SARS-CoV-2 weekly incidence rates for the number of PCR tests performed (Appendix, part B). For the period of regular school attendance (May–June), we compared weekly incidence rates during this time period with those of the week before school reopening (April 26–May 2). For summer vacation (July–August), we compared weekly incidence rates during this time period with those of the last week of school (June 21–27).

To determine positivity rate ratios (RRs) of SARS-CoV-2 tests, we calculated weekly average positivity RRs of SARS-CoV-2 PCR samples during school attendance and compared them with those of the week before school reopening (Appendix, part B). We calculated weekly average positivity RRs for summer vacation and compared them with those of the last week of school.

Because some children 3–9 years of age attended summer school during July, we compared the average weekly IRRs and positivity RRs of children 0–9 years of age and of children >10 years of age during August 2020 to those ratios from July. We reviewed time periods related to school closure and openings and mitigation measures used in schools (Appendix parts A, C). 

We studied the dynamics of slopes of the different variables across time periods as another approach for examining the involvement of children in COVID-19 spread during school attendance and summer vacation. We used linear regression analyses to compare the slopes of the adjusted weekly incidence of new cases in July–August (summer vacation) to those in May–June (school weeks) for each of the age groups. Because schools were reopened gradually in May 2020, we performed a separate analysis comparing the time period when all children attended schools (May 17–June 20, 2020) to slopes during summer vacation months (July–August 2020). We analyzed differences using 2-proportion z-tests and χ^2^ tests and used linear regression to generate the slopes. We used propagation of error statistics for measurements of uncertainties of slope RRs and slope arithmetic differences.

**Figure 1:**
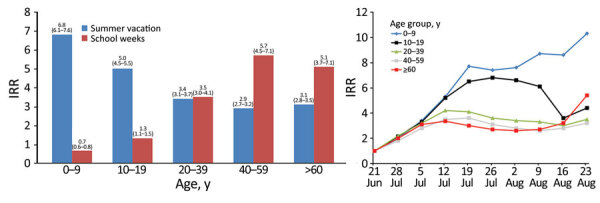
Mean weekly incidence rate ratios for COVID-19 during school attendance and during summer vacation, by age group, Israel, 2020. A) Adjusted IRR, the incidence adjusted for the number of COVID-19 tests performed for the specific age group. Numbers above bars are specific IRRs with 95% CIs. B) Weekly adjusted IRR during summer vacation by age group, which we calculated by comparing the incidence of each week to the incidence in the reference week for each age group. We calculated IRRs during school attendance by comparing the mean weekly rates during the school period (May–June), with rates during the week before school opening (April 26–May 3). We calculated mean weekly IRRs during summer vacation (July–August) by comparing the mean weekly rates during July–August to those of the last week of school (June 21–27), the reference week. Dates represent day 1 of the studied week. COVID-19, coronavirus disease; IRR, incidence rate ratio.

We observed higher IRRs in adults than in children during school attendance (May–June); the lowest increases were in the 0–9-year group (Figure 1, panel A). Mean positivity rates of tests performed in children 0–9 years of age during May–June was 0.012, compared with 0.04 in the week before school opening (positivity RR 0.3 [95% CI 0.24–0.36]). Mean corresponding positivity rate for those >10 years during May–June was 0.015, compared with 0.012 in the week before school opening (positivity RR 1.2 [95% CI 1.1−1.3]).

**Figure 2:**
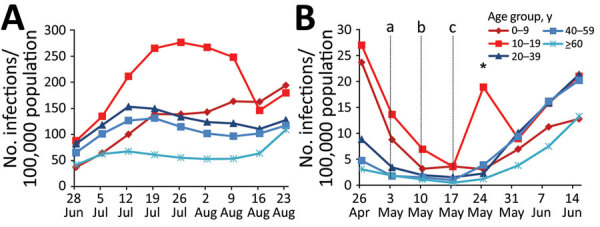
SARS-CoV-2 infection weekly adjusted incidence during school vacation (July 1– August 31) and attendance periods (May 3–June 19), Israel, 2020. Incidence was adjusted for the number of SARS-CoV-2 tests performed for the specific age group. Number of SARS-CoV-2 infections per 100,000 population for each age group are shown during summer vacation months (A) and during school weeks (B). Vertical lines represent partial reopening of schools (a), reopening of kindergartens and day care centers (b), and complete reopening of schools (c). Asterisk (*) indicates a single high school cluster. SARS-CoV-2, severe acute respiratory syndrome coronavirus 2.

In contrast, the 0–9 age group had the greatest increases in IRRs (Figure 1, panels A, B; Figure 2) and in positivity RRs (4.1 [95% CI 3.5–4.6] vs. 2.7 [95% CI 2.3–3.1] for older age groups combined). For children 0–9 years of age, we observed greater increases during August, when no schools were open, than during July, when some children 3–9 years of age attended summer schools, in IRRs (1.9 [95% CI 1.4–2.5]) and in positivity RRs of samples (1.7 [95% CI 1.6–1.8]). In contrast, for the other age groups combined, minimal increases were observed in IRRs (1.1 [95% CI 1.0–1.2]) and in positivity RRs (1.02 [95% CI 1.01−1.03]) (Appendix Figure 1).

We calculated slopes following linear regression of curves (Tables 1, 2; Appendix Table 2.). Children 0–9 years of age had the highest slope rate ratios and arithmetic differences when comparing SARS-CoV-2 adjusted incidence during summer vacation months with those of school weeks (May 3–June 29) (Table 1). Comparing the slopes from July–August to those obtained at the time when all children attended school (May 17–June 20) revealed that children 0–9 years of age had higher vacation/schools slopes rate ratio and higher arithmetic differences (Table 2).

**Table 1:** Comparison of slopes for adjusted incidence rates of SARS-CoV-2 infections during summer vacation and partial and full school attendance periods, Israel, 2020*

Age group, y	Adjusted incidence, %†		Slope rate ratio (95% CI)	Arithmetic difference (95% CI)
	Summer vacation ± SEM	School attendance ± SEM		
0–9	17.6 ± 2.1	1.8 ± 0.6	9.8 (5.3 to 34.2)	15.8 (10.9 to 21)
10–19	8 ± 8.8	3.4 ± 1.3	2.4 (–3.9 to 18.1)	4.7 (–15 to 24.4)
20–39	1.2 ± 2.9	4.5 ± 0.9	0.27 (–1.2 to 1.85)	–3.3 (–10.1 to 3.5)
40–59	2 ± 2.6	4.6 ± 1.8	0.43 (–8.9 to 3.6)	–2.6 (-8.9 to 3.6)
>60	4 ± 2.1	2.5 ± 0.6	1.6 (–0.2 to 4.5)	1.5 (–3.4 to 6.4)

*Summer vacation was July 1–August 31, 2020. School attendance period was May 3–June 28, 2020. SARS-CoV-2, severe acute respiratory syndrome coronavirus 2; SEM, standard error of the mean. †Incidence is no. cases/100,000 population; rates adjusted by numbers of SARS-CoV-2 tests.

**Table 2:** Comparison of slopes for adjusted incidence rates of SARS-CoV-2 infections during summer vacation weeks with those during full school attendance period, Israel, 2020*

Age, y	Adjusted incidence, %†		Slope rate ratio (95% CI)	Arithmetic difference (95% CI)
	Summer vacation ± SEM	School attendance ± SEM		
**0–9**	17.6 ± 2.1	2.6 ± 0.47	6.8 (4.3 to 11.7)	15 (8.7 to 21.3)
**10–19**	8 ± 8.8	3.2 ± 1.9	2.5 (–3.1 to 8.1)	4.8 (–14.2 to 20.8)
**20–39**	1.2 ± 2.9	5.3 ± 0.6	0.23 (–0.99 to 1.5)	–4.1 (–12.8 to 4.7)
**40–59**	2 ± 2.6	5.1 ± 0.34	0.4 (0.7 to 1.5)	–3.1 (–10.9 to 4.7)
**>**60	4 ± 2.1	3.2 ± 0.55	1.25 (–0.2 to 3.5)	0.8 (–5.5 to 7.1)

*Summer vacation was July 1–August 31, 2020. Full school attendance period was May 17–June 19, 2020. SARS-CoV-2, severe acute respiratory syndrome coronavirus 2; SEM, standard error of the mean. †Incidence rates adjusted by numbers of SARS-CoV-2 tests;

## Conclusions

The relative increase in SARS-CoV-2 cases and in positivity of samples during summer vacation was most prominent in young children 0–9 years of age, in sharp contrast to the period of school attendance, when it was relatively flat. Parallel trends among children aged 0–9 years during August compared with July indicate that the operation of summer schools during July did not significantly influence these results.

Analysis of curves of the adjusted incidence and comparisons of slopes of curves related to July–August to those for May–June revealed significantly higher rate ratios of slopes of adjusted incidence for children 0–9 years of age than any other age group. This analysis provides additional support to the finding that children in this age group are more likely to contract SARS-CoV-2 during vacation rather than during school attendance.

School closure has been shown to mitigate the spread of infection in conjunction with a lockdown (*3*). However, when schools are closed and a lockdown is not in effect, younger children tend to interact more intensively with adults than they do at school. Our findings suggest that school closure per se, without lockdown conditions, might not be sufficiently effective at reducing SARS-CoV-2 spread. We presume that because children may be less susceptible to COVID-19 infection and less infectious than adults, increased interaction with adults outside school may expose them more to SARS-CoV-2 infection (*4*–*6*). In addition, children may contract infection during regular and casual social encounters outside schools. The increased IRRs among adults during school period without a precedent or parallel increase in children suggest that SARS-CoV-2 transmission in children during school attendance does not necessarily lead to substantial increases in community transmission. In addition, in the described scenario, schools were closed because the academic year ended and not as a response to increased SARS-CoV-2 spread.

Our findings contrast with those seen in influenza epidemics, in which children play a leading role (*7*,*8*). A recent report suggests that contacts outside school were associated with SARS-CoV-2 infection, whereas attending school or child care was not associated with having positive SARS-CoV-2 test results (*9*). We also published a recent study that suggested that school reopening during May 2020 had a limited effect on COVID-19 spread in Israel (*10*). Those findings are in accordance with the results of this study and may be relevant regarding school reopening, which has been a challenge in many countries (*11*,*12*).

The main limitation of our study is its observational design. However, this study relies on a solid national database, and its findings are consistent when examined by several methods and several parameters. The highly consistent finding with different comparisons of regressions (slope rate ratios and arithmetic differences between slopes), as well as with different parameters (adjusted incidence and positivity rates of tests), when taken together, strengthen the reliability of the results.

In conclusion, our results suggest that children, especially those <10 years of age, may contract SARS-CoV-2 infections mainly outside of school. The main implication of our findings is that that school closure without lockdown conditions might not be sufficiently effective in reducing SARS-CoV-2 spread.

Appendix Additional information about coronavirus disease spread during summer vacation, Israel.
