# Supplementary material for: Coronavirus Disease Spread during Summer Vacation, Israel, 2020
Source: Emerg Infect Dis. 2022 Jan;28(1):188–91. doi: 10.3201/eid2801.210177 (PMC8714207; doi:10.3201/eid2801.210177)
Supplement: Appendix — Additional information about coronavirus disease spread during summer vacation, Israel. [file 21-0177-Techapp-s1.pdf]

Article DOI: https://doi.org/10.3201/eid2801.210177

Coronavirus Disease Spread during Summer Vacation, Israel, 2020

Appendix

Part A: Time Periods Related to School Closure and Openings

Data on school closure and reopening were obtained from official publications of the Ministry of Health and public resources 1,2.

March 14–May 2, 2020: Complete school closure period. All schools closed during the initial stage of the COVID-19 pandemic.

May 3, 2020: Partial school reopening for children in grades 1–3 and 11–12.

May 10, 2020: Reopening of kindergartens and day care centers.

May 17–June 18, 2020: Complete open school period. Complete reopening of all schools.

June 19, 2020: End of the academic year for high schools (grades 7–12).

June 30, 2020: End of the academic year for elementary schools (grades 1–6).

June 19–August 31, 2020: High school summer vacation.

July 1, 2020: Start of summer school programs for some children 3–9 years of age.

August 1–August 31, 2020: Summer vacation continued, no activities of summer schools.

September 1: Start of new academic year.

Part B: Computation of Adjusted Incidence, Adjusted Incidence Rate Ratio (IRR), and Positivity Rate Ratio (RR)

We used the following definitions to analyze our data.

Adjusted Incidence

Severe acute respiratory syndrome coronavirus 2 (SARS-CoV-2) age-specific weekly incidence rates were calculated and adjusted for the number of tests performed for the following age groups: 0–9, 10–19, 20–39, 40–59 and $\geq$60 years. For each age group, incidence rate (weekly number of new cases /100,000 population of the specific age group) was multiplied by the proportion of this age group in the general population and divided by the proportion of the weekly number of samples taken from persons of this age group out of all samples.

IRR and RR for School Attendance Period

A: Mean weekly adjusted incidence of severe acute respiratory syndrome coronavirus 2 (SARS-CoV-2) or number of tests performed in each age group during the period of regular school attendance (May–June 2020).

A1: Mean weekly SARS-CoV-2 positivity rates of samples tested in each age group during the period of regular school attendance (May–June, 2020).

a: Weekly SARS-CoV-2 adjusted incidence in each age group during the week prior to school reopening (April 26–May 2, 2020).

a1: Weekly SARS-CoV-2 positivity rates of samples tested in each age group during the week prior to school (April 26–May 2, 2020).

IRR = A/a,

RR = A1/a1.

IRR and RR for Summer Vacation Period

A: Mean weekly SARS-CoV-2 adjusted incidence (for number of tests performed) in each age group during the period of summer vacation (July–August, 2020).

A1: Mean weekly SARS-CoV-2 positivity rates of samples tested in each age group during the period of summer vacation (July–August, 2020).

a: Weekly SARS-CoV-2 adjusted incidence in each age group during the last week of school (June 21–27, 2020).

a1: Weekly SARS-CoV-2 positivity rates of samples tested in each age group during the last week of school (June 21–27, 2020).

IRR = A/a,

RR = A1/a1.

Part C: Mitigation Measures during School Attendance

The following are the guidelines provided to schools and parents regarding reopening, May–June 2020 (*1*).

Masks

Children >7 years of age were required to wear masks in classrooms and in public areas (*2,3*). However, between May 19–22 2020, restrictions on wearing masks in classrooms and open space were lifted due to a heat wave.

Daily Screening

Parents filled in and signed a daily report of their child's health including body temperature, as a prerequisite for school attendance. There were no recommendations for screening of teachers unless they were exposed to a confirmed SARS-CoV-2 case.

Social Distancing

According to national policies, the entrance of pupils to schools should be controlled to prevent crowding. It was recommended that activities during recess should be separated between classes. However, the degree of implementation of these measures was unclear.

During the first stage of school reopening (on May 3, 2020), the children were divided to separate "capsules" that were supposed to study in different times and different days, however these restrictions were lifted on May 17 and schools were completely reopened.

Taking Examinations

In addition, specific recommendations were provided for taking examinations (*4*).

Quarantine

Classmates and teachers of SARS-CoV-2 infected pupils or teachers were required to stay in quarantine and were also screened for SARS-CoV-2 infection regardless of whether they were symptomatic or asymptomatic.

School Closure

Temporary school closing was recommended if $\geq$3 cases of SARS-CoV-2 infection were identified in persons attending school.

Part D: Regression Analyses of Curves

Randomness

All analyzed data were based on Israeli Ministry of Health reports of new SARS-CoV-2 infections without selection or elimination. The Ministry of Health has operated a Coronavirus Surveillance Computerized Database including the results of RT-PCR tests and epidemiological investigations with mandatory reporting of laboratory-confirmed SARS-CoV-2 infections and associated hospitalizations.

Sample Size

We have provided samples from the specific age groups that were used for creating the curves and calculating regression analyses (Appendix Table 1) and linear regression parameters (Appendix Table 2).

References

1. Coronavirus and Schools in Israel. Information and Research Center of the Israeli Knesset, June 21, 2021 [cited 2021 Nov 19]. https://main.knesset.gov.il/Activity/Info/mmm/pages/document.aspx?docid=A132460F-7682-EB11-810D-00155D0AEE38

<eref>2. The Hebrew University, Jerusalem, Israel, July 31, 2020 [cited 2021 Nov 19]. https://new.huji.ac.il/sites/default/files/mainsite/files/huji_3107.pdf

<eref>3. Guidelines of the Ministry of Education, Israel, August 31, 2021 [cited 2021 Nov 19]. https://meyda.education.gov.il/files/Bitachon/SchoolcoronarulesofconductFullRoutine.pdf

4. Taking Examination during Coronavirus Pandemic: Information and Research Center of the Israeli Knesset. October 26, 2020 [cited 2021 Nov 19]. https://main.knesset.gov.il/activity/info/mmm/pages/document.aspx?ver=2&docid=7cf27135-c516-eb11-8108-00155d0aee38

Appendix Table 1. Positive samples and total number of tests used for regression analysis in study of coronavirus disease incidence during school session and vacation, Israel, 2020*

		Full school attendance period		Summer vacation	
Age group, y	No. in age group	No. tests	Positive tests	No. tests	Positive tests
0–9	1,719,000	37,735	395	141,592	9,777
10–19	1,428,000	50,585	807	158,510	16,859
20–39	2,380,000	109,437	2066	444,969	32,518
40–59	1,818,000	66,673	887	333,897	20,870
≥60	1,367,000	68,908	449	303,859	10,911

*Full school attendance period was May 17–June 19, 2020; summer vacation was July 1–August 31, 2020.

Appendix Table 2. Linear regression parameters for adjusted incidence curves in different time periods analysis in study of coronavirus disease incidence during school session and vacation, Israel, 2020*

Age group, y	Regression line equation	Regression coefficient	Regression coefficient error	Standard error	p value†	R^2	p value‡
School attendance period							
0–9	y = 15+ 2.6x ± 1.4	2.64	0.47	1.4	0.01	0.91	reference
10–19	y = 23 + 3.2x ± 6.02	3.17	1.9	6.02	0.19	0.48	0.49
20–39	y = 26 + 5.3x ± 1.9	5.32	0.6	1.9	0.0003	0.96	0.0080
40–59	y = 25 + 5.1x ± 1.13	5.1	0.35	1.13	0.00076	0.98	0.0034
≥60	y = 14.8 + 3.2x ± 1.7	3.2	0.54	1.7	0.01	0.92	0.11
Summer vacation							
0–9	y = 215 + 17.6x ± 14.5	17.6	2.1	14.5	0.0004	0.92	reference
10–19	y = 241 + 7.97x ± 61.2	7.97	8.9	61.2	0.29	0.10	0.05
20–39	y = 130.1 + 1.18x ± 14.3	1.18	2.89	14.3	0.11	0.10	0.0008
40–59	y = 116 + 2.05x ± 13.4	2.05	2.6	13.4	0.19	0.62	0.0004
≥60	y = 82.8 + 3.98x ± 17.7	3.98	2.14	17.7	0.13	0.33	0.0001

*Full school attendance period was May 17–June 19, 2020; summer vacation was July 1–August 31, 2020.
†p value of the specific regression.
‡p value of the regression coefficient of the specific age group compared to that of children 0–9 years of age.

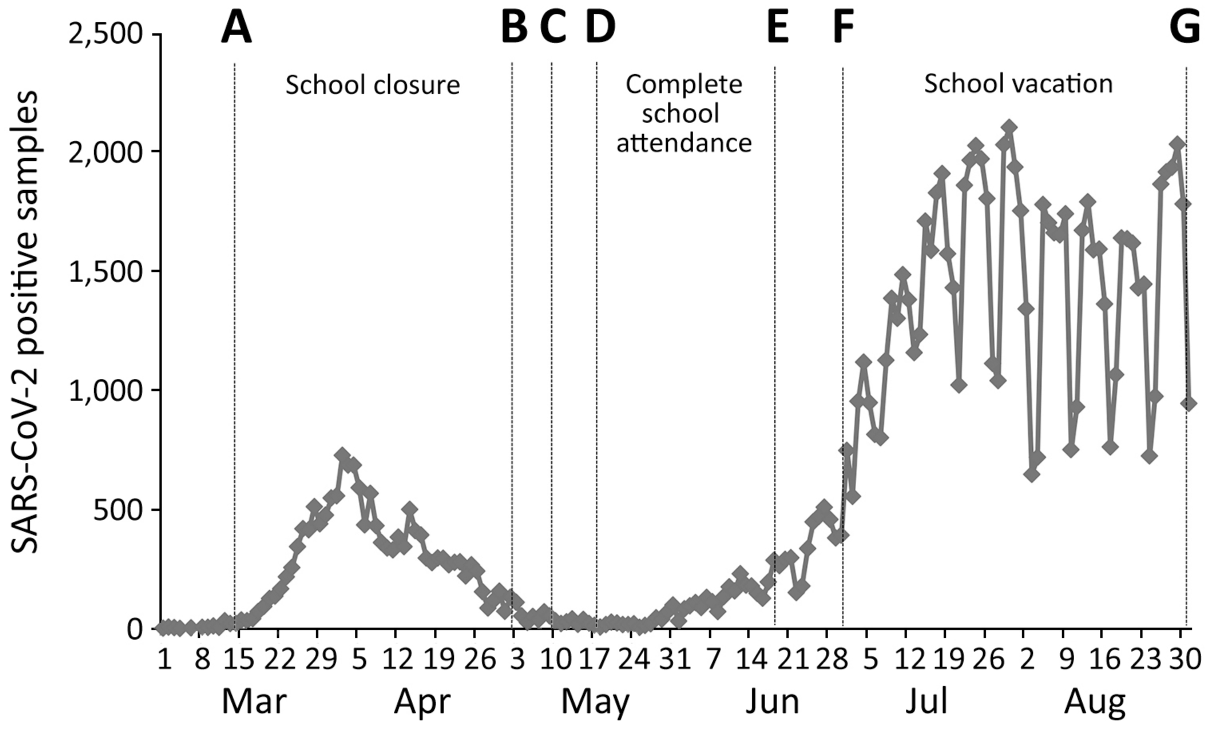

Appendix Figure. SARS-CoV-2 daily incidence in the general population, Israel. Numbers of daily new SARS-CoV-2 cases during March 1–August 31, 2020 are shown. Time points related to school closure, school opening and summer vacation are indicated.
